# Radon Risk and Remediation: A Psychological Perspective

**DOI:** 10.3389/fpubh.2017.00063

**Published:** 2017-03-27

**Authors:** David Hevey

**Affiliations:** ^1^Research Centre for Psychological Health, School of Psychology, Trinity College Dublin, Dublin, Ireland

**Keywords:** radon, risk perceptions, psychology, decision-making, threat perception

## Abstract

Although radon exposure in the home increases the risk of lung cancer, this risk can be managed. However, evidence indicates that testing for radon and subsequent home remediation rates are generally low in many countries. The present perspective outlines some key insights from psychological science that might account for sub-optimal radon protection. Psychological aspects of how the health risks posed by radon are perceived and managed are outlined. There is need to consider radon risk perception in terms of the (a) cognitive and emotional responses to radon and (b) social context in which the radon threat occurs. In addition, the nature of the threat itself is integral to the failure for people to act in response to a radon threat. Finally, the challenges arising from defensive processing of radon threat information are outlined.

Lung cancer is the leading cause of cancer-related deaths worldwide. Following tobacco smoke, exposure to radon is the second leading cause of lung cancer in many countries, including the USA (1). The World Health Organization (WHO) notes that radon causes up to 14% of lung cancers worldwide (2). Each year, according to current estimates by the US EPA (3), approximately 21,000 lung cancer deaths in the United States are associated with radon exposure; in Canada, an estimated 3,000 deaths are associated with radon (4). Radon (Rn-222) is a colorless, tasteless, odorless natural radioactive noble gas that originates from the decay of uranium-238, a naturally occurring radioactive mineral found in the earth crust. The levels of radon gas can build up indoors, especially in the lower levels of a building. Once inhaled, the radioactive decay products of radon can adhere to cells lining the lungs thus exposing the sensitive bronchial epithelial cells to alpha radiation, which can lead to lung cancer. Radon levels in the home can be easily tested for and homes can be remediated to reduce the associated risk; however, the literature in general indicates low levels of radon testing and home remediation. Such low levels of testing are not exclusively caused by cost: when offered radon tests for free, less than 40% of the residents in an area with high radon levels availed of the offer (5). Many people underestimate the seriousness or long-term health effects of radon exposure. Furthermore, even when individuals are informed that their homes have high radon levels and are made aware of the consequent health threats, remediation rates are still low (6). A review comparing remediation rates in Ireland with other countries indicated high variability between countries; in general, only one in five remediate in response to dangerous levels of radon (7).

Comprehensive multi-media information programmes to increase radon testing and remediation internationally are successful at increasing awareness of radon; however, research consistently finds low levels of radon testing and remediation following such programmes (7–9). It is in this context that the present perspective examines the psychological aspects of how the health risks posed by radon are perceived and managed.

In many countries, radon testing and remediation are the responsibility of the individual: consequently, the goal of public awareness communications is to help individuals take appropriate preventive action. The individual is responsible for (i) testing to determine to what extent radon is present, (ii) deciding if the level poses a threat, (iii) selecting an appropriate remediation strategy, (iv) implementing the remediation strategy, and (v) retesting to ensure that remediation has been successful. To ensure that the individual has the requisite knowledge to make an informed decision regarding radon, the government’s role is typically to provide information to individuals regarding the threat radon poses, its assessment, and potential remediation strategies. The information provision approach is based on an assumption that individuals will act rationally in relation to the information provided; once you tell people that there is a threat, they will be motivated to test to see if they personally are at risk from the threat, they will test, and then they will act to remediate if the test indicates a threat. However, if we break down the process of translating the information into necessary behaviors, there are a number of stages that need to occur for an individual to act following an information programme (Table 1).

**Table 1:** **Steps required for action to occur after radon information programme**.

I am *exposed* to the information. I *attend* to the information (notice it). I am *interested* in the information. I *understand* the information. I *believe* that there is a threat: the information must be perceived as being *credible*. The threat is *comprehensible*: I understand the threat. I *perceive* it as a possible *risk*: the threat may affect me (I may be *susceptible*) and it may have very negative health consequences for me (it is *severe*). I *believe* that the threat level can be assessed. I *know* how to get the threat level assessed. I *want* to get the threat level assessed. I *act* to get the threat level assessed: test. I *understand* the results. I *perceive* that *I am at risk* (I am susceptible to a severe negative outcome). I *want* to reduce this risk. I *know* how to reduce this risk. I *act* to reduce the risk: remediate. I *act* to confirm that the risk has been managed: re-test.

The above list, which arguably is an over-simplification of the process, comprises a mixture of the awareness programme content and the individual’s perceptions, knowledge, motivation, and actual behavior. A number of steps need to occur before an individual will test and then remediate. This sequence is predicated on the assumption of a rational actor responding to health threat information, i.e., an informed individual tends to behave in the best interests of their health (10). However, such an assumption does not fit with psychological research on risk perception and risk-reducing behaviors: a key theme in this paper is that people can respond to health threat information in a sub-rational manner, and that such responses reflect both powerful unconscious and deliberate psychological processes. In order to better understand the failures of individuals to act to assess and then remediate against the threat from radon, the psychology of risk perception needs to be considered.

## Risk Perception

How the individual perceives the risk of radon determines decision-making regarding radon testing and remediation. Perceived risk is associated with both intentions to test as well as actual radon test ordering (11). In addition, among those in areas of high radon levels, the perception of radon as a health risk is related to intentions to conduct radon testing and remediation (12). Although risk from an epidemiological perspective broadly refers to a quantitative measure of the probability of experiencing some negative outcome, risk from a psychological perspective is a far more complex and nuanced construct. For example, risk perception can be defined as “people’s beliefs, attitudes, judgments, and feelings, as well as the wider social or cultural values and dispositions that people adopt, toward hazards and their benefits” (13). This definition explicitly highlights the inherent complex multidimensional (cognitive and emotional responses) and context-specific (e.g., community, cultural and social values, and behaviors) aspects to risk perception. Consequently, we need to consider radon risk perception in terms of the (a) cognitive and emotional responses to radon, and (b) social context in which the radon threat occurs.

### Cognitive and Emotional Responses to Radon

In the rational actor approach, individuals should process health threat information in an objective and considered manner, and this appraisal of threat will determine their behaviors in response to being made aware of the threat. Psychological theory and research regarding how people actually respond to health threats such as radon paints a more complex picture of how we process health threat information. A number of well-established cognitive heuristics (“mental shortcuts”) (14, 15) impact on our risk perception of radon, which impede appropriate behavioral responses.

*Availability* refers to our tendency to judge the likelihood of future events, such as developing lung cancer due to radon, based on how easy it is to imagine them or to recall similar events in memory. In general, if people cannot recall either someone developing lung cancer due to radon, then such examples are not available to form a judgment that radon is a risk to one’s health. Although people can recall hearing of radon, how many will be able to recall someone developing or dying from lung cancer due to it? In the absence of such associations, the risk can be downplayed or ignored in the majority of the population.

*Representativeness* refers to how individuals make judgments about the likelihood of an event based on its resemblance to their past experiences or assumptions. It reflects a principal means by which judgments are made: whether or not something is a member of a broader category. For example, smoking is commonly accepted as a member of a broader category of things that are risks for lung cancer. Similarly, someone getting lung cancer after a period living next to a nuclear plant fits within these assumptions; however, in the context of radon, getting lung cancer from simply being in one’s own home does not fit these assumptions and experiences. People tend to worry more about radiation from nuclear plants than radiation in their home; consequently, people negate the risk from radon in the home.

*Unrealistic optimism* occurs when individuals have unreasonably low estimates of their own susceptibility to harm. For example, people who did not test radon in a high risk area in the US held “optimistic biases” whereby they underestimated the risks associated with their own exposure to radon (16). Furthermore, such unrealistic optimism was present among respondents living in a very high radon area in Ireland; in essence, participants believed that radon was a threat to others in the community but not for themselves—hence, no need to test or worry about radon (17).

Our emotional response to a threat can influence on decisions regarding testing and remediation. For example, fear of cancer diagnosis and its symptoms and embarrassment are recurring themes in the research literature on barriers to attending cancer screening (18). Similar issues may contribute to the failure to test for radon. This issue is considered later in the context of defensive processing of threat information. Of note, individuals feel more threatened by a description of radon that assigns radon agency (19): people are more worried by radon that is described as deliberately targeting a home (e.g., “Radon gas invades people’s homes”) than a literal description of radon dissemination into a home (e.g., “Radon gas seeps into people’s homes”). Assigning agency to radon primes an emotional response to the threat to our home and sense of security. We have an emotional identification with our homes: consequently, it is hard to accept that our home (our physical and psychological place of safety and security) is a threat to our health.

### Features of Radon

In general, a core challenge for communicating radon risk and promoting radon remediation relates to the fact that *radon threat is inherently perceived as either being low or simply non-existent* (5, 9). The nature of radon and its threat level serve to minimize an urgency to act accordingly. Radon is a colorless, odorless, and tasteless gas. Consequently, there is an absence of sensory cues to alert people to the risk: such cues to action typically help motivate behavior (20). Radon does not seem to cause any visible health effects and in the absence of sensory cues the risk is, in essence, out of mind. The risk from radon is natural: in general, we perceive technological threats to be more risky than natural threats (21). It is arguable that if the lung cancer rate caused by the natural process of radon emission was associated with a manufactured process, there would be widespread outrage and immediate calls for governmental action.

Overall, the level of risk associated with radon is perceived as being so low that the risk is not understood or appropriately acted upon. In general, for low levels of risk, people can easily dismiss the risk as being too small to worry about (22) as we do not see it as being likely to happen to ourselves (unrealistic optimism also influences this judgment). The experience of the radon risk is benign as people live with the risk, sometimes for many decades, without experiencing any side effects or symptoms. In addition, the effect of the risk is far removed from the initial exposure: the lung cancer will develop decades later and, as there are no early symptoms to act as cues, it is easy to delay action.

### Social Context to Radon Threat

For individuals in a community, risk perception is informed by a wider framing of the issue, derived from their personal experiences in a given context, including how their interpersonal networks respond to the risk (23). Risk perceptions are affected by the norms of the groups with which people identify. In essence, lay risk perception is based on a wider framing of topics, considerations, and agendas. Risks are shared and experienced collectively. People look to their social networks for information and guidance, particularly their trusted sources. In terms of radon, this implies that if there is no collective action in relation to radon testing and remediation then the individual may not perceive it as being a threat to be concerned about. Indeed, such social norms influenced attitudes toward testing in a particularly high radon area (where one house had radon levels 245 times the national reference level for Ireland): individuals reported that as others in the community did not test, then themselves would not test for radon (17). In summary, radon risk perception reflects personal experiences and circumstances and is highly influenced by social context.

## Defensive Risk Information Processing

At a basic level, how we process risk information can result in our not taking appropriate prevention action. Exposure to a health threat communication initiates two appraisal processes: threat appraisal and coping appraisal (24). Individuals will appraise the threat portrayed in the communication, and the more they believe they are vulnerable to a serious threat, the more motivated they will be to engage in coping appraisal. However, if the radon threat is perceived as irrelevant (“It affects other people’s houses, not mine”) or insignificant (“The threat is so low”), then there is no motivation to process the radon information any further, and individuals will simply ignore the remainder of a communication. As noted previously, unrealistic optimism and the challenges of understanding low probabilities can act to minimize our sense of susceptibility and severity to the radon threat. In addition, people believe that they can, at a later point, undo any radon-related damage they have done to themselves by inaction at present or in the past (25)—which further serves to reduce the need to remediate immediately as the severity of the threat can “minimized” through later action.

When the radon risk is believed to be serious and relevant, individuals will become scared, and their fear should motivate them to consider their coping alternatives. However, research evidence indicates that *the more personally significant* a health message is, the more people are likely to downplay the seriousness of the health risk, question the accuracy of the threatening information or evidence presented in the message, and process the information in a biased fashion (26–28). The main audience of radon messages is the at-risk population (i.e., those who live in an area with high levels of radon), but these people may also the most difficult to persuade because they often defensively process the information as it is too threatening (“You are at risk of developing lung cancer due to radon”). When presented with such a message, an individual can engage in defensive mechanisms that function to reduce the threat (29, 30). People mostly at risk, those for whom the message is most personally relevant, are typically the ones most likely to employ defensive techniques such as message avoidance (31) or denial of susceptibility (32). Increased personal relevance affects the type of processing used and subsequent evaluation of message information (27, 28). For example, “defensive systematic processing” characterizes how those individuals at risk are more critical of portions of the persuasive messages linking their behavior with a threat and less critical of the portions of the message that shed doubt on that link (28). For example, an individual will actively try hard to question the evidence for the relationship between radon and lung cancer, but will devote less cognitive effort to evaluate a statement that radon is an odorless gas. Individuals can process information systematically with a bias toward information that maintains the current *status quo*, which will inhibit their behavioral responses to actually test or remediate.

## Summary

Risk has a cognitive aspect (i.e., what we know about the risk) and an emotional aspects (i.e., what we feel in terms of dread or fear about it). Until relatively recently health and environmental threat communications have tended to focus on the cognitive aspects (in the assumption that people are rational actors once provided with relevant information), whereas research consistently shows that individuals’ actions can be driven by the emotional aspects of risks and the need to manage the emotional threat to self. Information will only act as a driver of behavior only if it can overcome the numerous biases that individuals have toward processing risk information. When risks threaten, some cognitive and emotional mechanisms push people toward action; others push them toward inaction. The threat from radon can easily be downplayed to justify inaction.

Risk perception is a complex psychological process of meaning-making by the individual; it is subject to numerous unconscious, cognitive, and emotional biases that influence how we process radon information. These biases act to minimize our sense of risk. Given these challenges, it is not surprising that radon threats fail to promote appropriate precautionary behavior. Even where there is awareness of radon, apathy rather than a sense of urgency tends to be reported (16). There are no immediacy markers of threat: there are no obvious “dead bodies,” and the radon-related lung cancer occurs in the distal future (33).

A multidisciplinary approach, involving ongoing collaboration with experts from the field of psychology, has been advocated as essential to solve the problems associated with a lack of radon remediation (34). A core challenge for risk awareness programmes is to inform the target audience in ways that do not create undue apathy, complacency, or overconfidence while also not creating undue stress or alarm (35). For radon, this is quite a complex challenge, as the risk is perceived as distal, uncertain, and easily discounted. The present paper has highlighted some of the challenges that risk communicators must address to enhance radon testing and remediation rates.

## Author Contributions

DH conducted the review of the literature and wrote the manuscript.

## Conflict of Interest Statement

The author declares that the research was conducted in the absence of any commercial or financial relationships that could be construed as a potential conflict of interest.
